# Anticoagulation for Stroke Prevention in Patients with Atrial Fibrillation: A Review of the Literature and Current Guidelines

**DOI:** 10.31083/RCM39233

**Published:** 2025-06-26

**Authors:** Vrinda Vyas, Vandita Vyas, Akash Sharma, Prashanth Ashok Kumar

**Affiliations:** ^1^Cardiology, ECU Health, Tarboro, NC 27886, USA; ^2^Anesthesiogy and Intensive Medicine/Critical Care, Marienhospital, 70199 Stuttgart, Germany; ^3^Department of Medicine, University at Buffalo – Catholic Health System, Buffalo, NY 14214, USA; ^4^Hematology-Oncology, George Washington University, Washington, D.C. 20037, USA

**Keywords:** atrial fibrillation, anticoagulation, hemorrhage, stroke

## Abstract

Atrial fibrillation (AF) is the most common arrhythmia worldwide, characterized by uncoordinated atrial activation leading to a loss of effective atrial contraction and increased risk for atrial thrombi formation, promoting an increased risk of cardioembolic strokes and mortality, and associated increased healthcare expenditure. Therefore, stroke prevention represents a key focus in managing patients with atrial fibrillation, and strategies to achieve this aim have drastically evolved over the years. Previously, aspirin and warfarin were the cornerstone of stroke prophylaxis. However, direct oral anticoagulants have emerged and are now recognized as a safer and more effective alternative for non-valvular AF. Meanwhile, newer non-pharmacological methods to prevent AF related strokes, such as left atrial appendage occlusion devices, have been approved to ameliorate the need for lifelong anticoagulation in patients with elevated bleeding risks. This review outlines the current recommendations and provides an overview of the literature on stroke prevention in patients with atrial fibrillation, particularly focusing on using direct-acting oral anticoagulants. Comparisons between these agents and special considerations for use are also reviewed.

## 1. Introduction

Atrial fibrillation (AF) is the most common heart rhythm disorder with an increasing incidence and prevalence across the world [1, 2]. In 2020, the estimated global prevalence of AF was around 50 million [2, 3]. In 2010, the prevalence of AF in the United States was estimated at 5.2 million, with projections indicating a threefold increase by 2030 [4]. AF also contributes to substantial morbidity and mortality; it has been shown to nearly double the risk of death, increase the risk of stroke by 2.4 times [5], double the risk of sudden cardiac death [6], and raise the risk of heart failure (HF) by five times [5]. A study found that the most common outcomes associated with an AF diagnosis included death (48.8% at five years), HF (13.7%), new-onset stroke (7.1%), and gastrointestinal bleeding (5.7%) [7]. Consequently, AF is linked to substantially higher healthcare expenditure and was responsible for $28.4 billion in healthcare costs in 2016 alone [8].

**Fig. 1.**
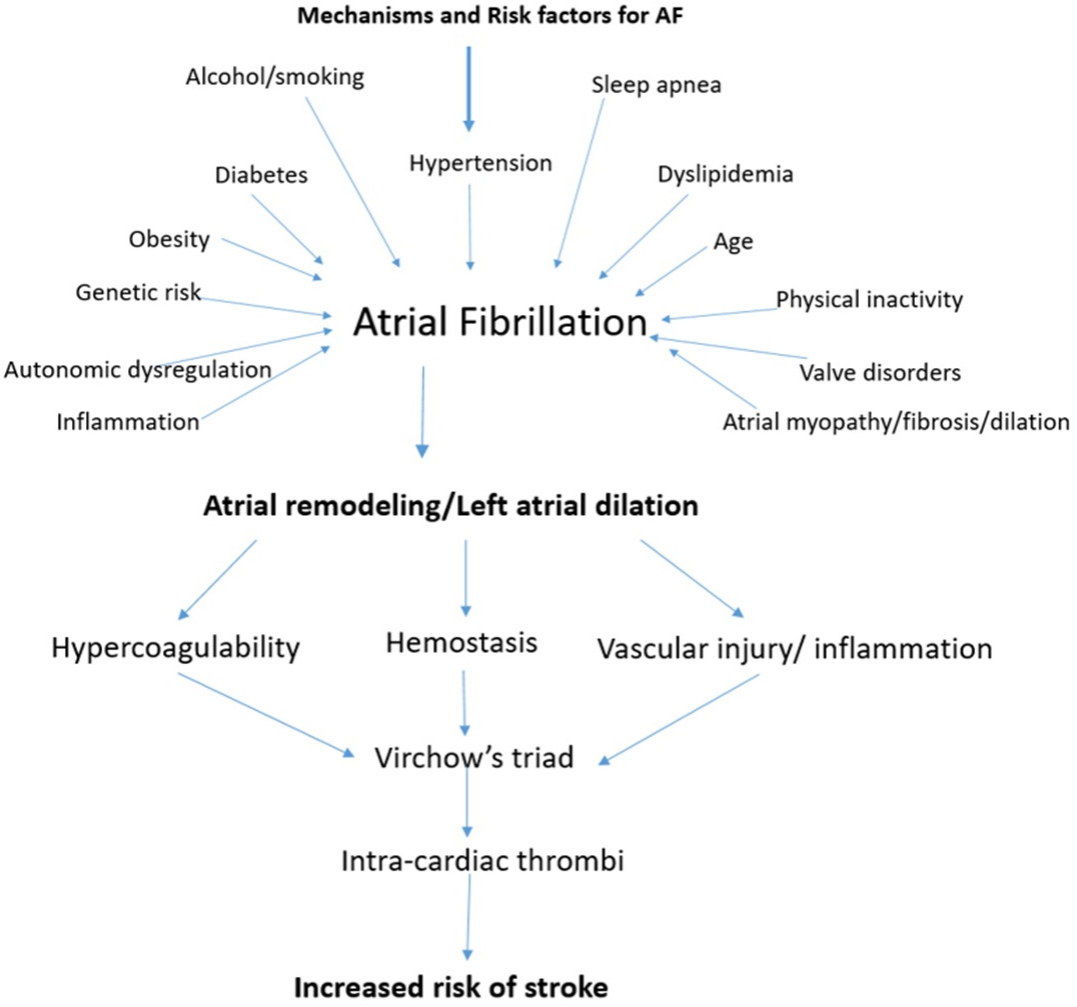
**The Pathophysiology of Stroke in atrial fibrillation (AF)**.

AF is characterized by an irregular atrial rhythm leading to irregular activation of the ventricles, diagnosed on the electrocardiogram (ECG) by the absence of well-defined P waves and varying R-R intervals. Normal cardiac conduction involves impulse initiation by the sinoatrial node, which then conducts uniformly across the atria to the atrioventricular node and beyond. AF occurs as a result of ectopic potentials usually generated by the pulmonary veins or secondary to reentrant activity caused by interstitial fibrosis within the atrial tissue [9, 10]. Atrial myopathy is increasingly being recognized as the structural and/or electrophysiological abnormalities occurring within the atrial tissue as a result of interaction of inflammatory stressors, autonomic dysregulation, oxidative stress, atrial stretching and fibrosis. Atrial myopathy then facilitates the rapid and irregular impulse origination and conduction which is characteristic of AF. The interaction between these mechanisms perpetuates a vicious cycle, leading to progressive atrial myopathy and a heightened risk of persistent AF [11]. As a result of this sustained erratic electrical activity, there occurs a state of increased hemostasis within the left atrium, which further leads to endothelial dysfunction and hypercoagulability. The left atrial appendage (LAA) is a muscular, blind-ended pouch extending from the left atrium. Progressive atrial myopathy associated with AF enhances the thrombogenic potential of the LAA. Its complex morphology—characterized by a narrow orifice, variable lobes, and extensive trabeculations—predisposes to significant hemostasis, as evidenced by reduced LAA peak flow velocities, thereby facilitating thrombus formation. Consequently, the LAA is the site of thrombus formation in approximately 90% of patients with non-rheumatic AF [12]. The thrombotic material can then embolize to the cerebral circulation, leading to strokes [13]. In one study, AF was associated with a fivefold increased risk of stroke, with an estimate suggesting 20% of all strokes being linked to AF [14]. To reduce the risk of embolic strokes, oral anticoagulation (OAC) has long been a key component of treatment for AF. Fig. 1 summarizes the pathophysiology of atrial fibrillation related stroke.

Risk stratification tools have been developed to help guide anticoagulation treatment strategies in clinical AF. CHADS_2_ score was traditionally used to assess stroke risk (with points for chronic heart failure, hypertension, age, diabetes, and 2 points for prior stroke/transient ischemic attack (TIA). Many of these scores only have modest predictive value, discrimination, and lack correlation with real world outcomes because they fail to account for additional factors that may influence stroke risk, particularly the specific AF attributes for an individual patient [13, 14, 15]. These scores also do not take into account other comorbid conditions increasing thromboembolic risk such as presence of cancer, obesity, smoking status, and chronic kidney disease (CKD) [16, 17]. One of the most popular risk scores is the CHA_2_DS_2_-VASc score, with 1 point for HF, 1 point for high blood pressure, 2 points for age ≥75 years, 1 point for age between 65–74 years, 1 point for diabetes, 2 points for prior cerebrovascular accident, 1 point for vascular disease, and 1 for female sex. Other risk scores such as CHA_2_DS_2_-VASc–R (R as African American), R_2_ (creatinine)- CHADS_2_, and ATRIA Stroke Risk Score have also been developed [18, 19, 20]. Being a female is now considered a “risk-modifying factor” rather than a true independent risk factor for AF related stroke [21, 22]. To eliminate sex as a risk factor, ESC recommends CHA_2_DS_2_-VA score which excludes birth sex as a risk factor, whereas ACC/AHA guidelines still recommend CHA_2_DS_2_-VASc score. A summary of various commonly used models for stroke risk assessment in AF is provided in Table 1 (Ref. [15, 16, 17, 18, 23]).

**Table 1.** **Stroke risk assessment scores for patients with AF**.

Score name	Components and corresponding points	Interpretation of scores	Stroke risk (%)
CHADS_2_ [15]	Heart Failure (1), Hypertension (1), Age ≥75 (1), Diabetes (1), Prior Stroke/Systemic Embolism (2)	0 = Low risk, 1–2 = Moderate risk, ≥3 = High risk	0 = 0.5%, 1 = 1.3%, 2 = 2.2%, 3 = 3.2%, 4 = 4.0%, 5 = 6.7%, 6 = 11.2%
CHA_2_DS_2_-VASc [16]	Heart Failure (1), Hypertension (1), Age 65–74 (1) / ≥75 (2), Diabetes (1), Prior Stroke (2), Vascular Disease (1), Female (1)	0 = Low risk, 1 = Moderate risk, ≥2 = High risk	0 = 0.3%, 1 = 0.9%, 2 = 2.2%, 3 = 3.2%, 4 = 4.8%, 5 = 7.2%, 6 = 9.7%, 7 = 11.2%, 8 = 10.8%, 9 = 12.2%
CHA_2_DS_2_-VASc–R [23]	Heart Failure (1), Hypertension (1), Age 65–74 (1) / ≥75 (2), Diabetes (1), Prior Stroke (2), Vascular Disease (1), Female (1), Race (1)	0 = Low risk, 1 = Moderate risk, ≥2 = High risk	Similar to CHA_2_DS_2_-VASc, but refined for race
R_2_ -CHADS_2_ [17]	Heart Failure (1), Hypertension (1), Age ≥75 (1), Diabetes (1), Prior Stroke (2), Chronic Kidney Disease (eGFR <60) (2)	0 = Low risk, 1–2 = Moderate risk, ≥3 = High risk	0 = 0.5%, 1 = 1.6%, 2 = 2.2%, 3 = 3.7%, 4 = 5.9%, 5 = 9.0%, 6 = 11.2%
ATRIA [18]	Heart Failure (1), Hypertension (1), Age (0–6 no prior stroke/7–9 with prior stroke), Diabetes (1), Chronic Kidney Disease (eGFR <45 or ESRD) (1), Female (1)	0–5 = Low risk, 6 = Moderate risk, ≥7 = High risk	Low = <1%, Moderate = 1–3%, High = >3%

ATRIA, Anticoagulation and Risk Factors in Atrial Fibrillation Score; eGFR, estimated glomerular filtration rate; ESRD, end-stage renal disease.

As expected, anticoagulation while preventing thromboembolic strokes, simultaneously increases the risk of bleeding, hence, patients with AF also have to be assessed for bleeding risk. Commonly used bleeding risk scores include HAS-BLED (which involves scores for high blood pressure, abnormal renal/liver function, stroke history, bleeding history, labile international normalized ratio (INR), age ≥65 years, active use of certain drugs which increase bleeding risk), HEMORR_2_HAGES and ATRIA Bleeding Risk Scores. A summary of these scores is in Table 2 (Ref. [19, 20, 21, 22]).

**Table 2.** **Bleeding risk assessment scores for patients with AF**.

Bleeding risk score	Components & corresponding points	Interpretation
HAS-BLED [19]	- Hypertension (systolic BP >160 mmHg) 1	0–1 points: Low risk (1.13 bleeds per 100 patient-years)
	-Abnormal renal (dialysis, transplant) 1	
	-Abnormal liver function (cirrhosis, liver disease) 1	2–3 points: Moderate risk (1.88 to 3.72 bleeds per 100 patient-years)
	- Stroke 1	
	- Bleeding history 1	4–5 points: High risk (8.7 to12.5 bleeds per 100 patients-years)
	- Labile INR (if on warfarin) 1	
	- Elderly (age >65) 1	>5 points: Very high risk
	- Drugs (antiplatelets, NSAIDs) 1	
	- Alcohol use (>8 drinks/ week) 1	
ATRIA [20]	- Age ≥75 years 2	<4 points: low risk (0.76% Annual Risk of Hemorrhage)
	- History of bleeding 1	4 points: intermediate risk
	- Anemia (Hb <13 g/dL men, <12 g/dL women) 3	>4 points: high risk (5.8% Annual Risk of Hemorrhage)
	- Renal impairment (eGFR <60 mL/min) 3	
	- Hypertension 1	
ORBIT [21]	- Age >74 years 1	0–2 points: Low risk (2.4 bleeds per 100 patient-years)
	- History of bleeding 2	3 points: Medium risk
	-Antiplatelet use 1	4–7: High risk (8.1 bleeds per 100 patient-years)
	- Anemia (Hb <13 g/dL men, <12 g/dL women) 2	
	- Renal disease (eGFR <60 mL/min) 1	
HEMORR_2_HAGES [22]	- Hepatic or renal disease 1	0–1 points: Low bleeding risk (1.9% to 2.5% risk of bleeding per 100 patient-years of warfarin)
	- Ethanol abuse 1	
	- Malignancy 1	2–3 points: Moderate bleeding risk (5.3% to 8.4% risk of bleeding per 100 patient-years of warfarin)
	- Older age (>75 years) 1	
	- Reduced platelet count or function 1	≥4 points: High bleeding risk (10.4% to 12.3% risk of bleeding per 100 patient-years of warfarin)
	- Rebleeding risk 2	
	- Hypertension (uncontrolled) 1	
	- Anemia 1	
	- Genetic factors 1	
	- Excessive fall risk 1	
	- Stroke 1	

BP, blood pressure; Hb, hemoglobin; INR, international normalized ratio; NSAIDs, nonsteroidal anti-inflammatory drugs.

**Fig. 2.**
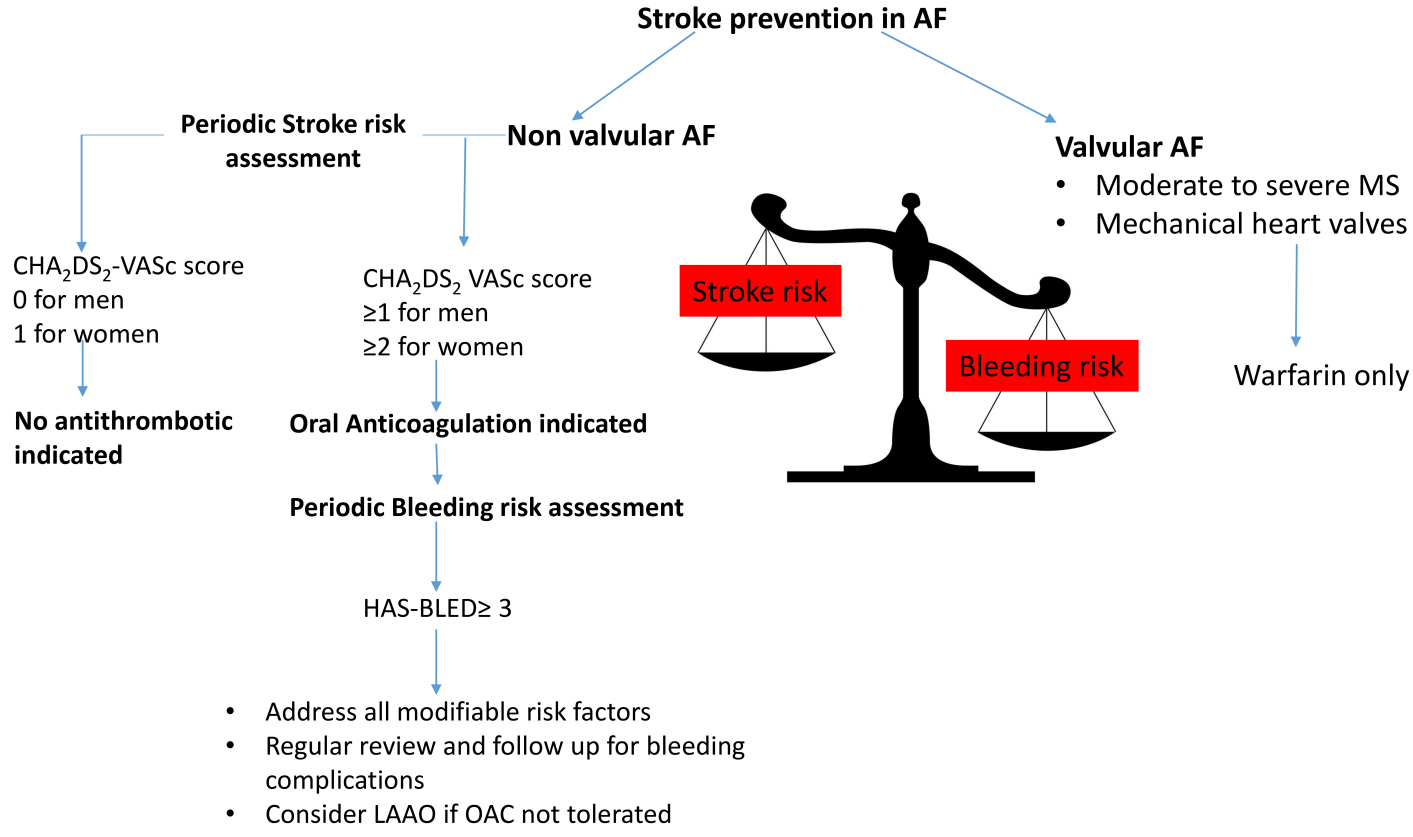
**Approach to oral anticoagulation in atrial fibrillation**. LAAO, left atrial appendage occlusion.

Similar to the stroke risk scores, these bleeding risk scores also have poor discrimination. These scores incorporate several factors that not only predict a higher stroke risk but also an increased risk of bleeding (such as hypertension, stroke, kidney disease, and age) thereby confounding the application of bleeding risk scores for individual patients. This review provides an overview of contemporary strategies for anticoagulation in stroke prevention for AF, with a particular emphasis on the current European Society of Cardiology (ESC) and American College of Cardiology (ACC)/American Heart Association (AHA) guidelines. Fig. 2 shows the Approach to Oral Anticoagulation in AF.

## 2. Antiplatelets

A comprehensive analysis of clinical trials focused on AF has established that while aspirin diminishes the likelihood of thromboembolic strokes when compared to a placebo, it is less effective than warfarin [24, 25]. The AVERROES trial highlighted the superiority of apixaban over aspirin in preventing strokes or systemic embolisms, revealing a significant reduction in risk associated with apixaban (hazard ratio, HR = 0.45; 95% CI: 0.32–0.62; *p* < 0.001). There was no notable difference in major bleeding events between the two treatment groups [26]. Consequently, aspirin is not regarded as a reasonable substitute for oral anticoagulation in the context of stroke prevention [27, 28]. Moreover, using antiplatelets can lead to negative outcomes, especially among older patients with AF [29, 30].

The combination of aspirin (75–100 mg daily) and clopidogrel (75 mg daily) offers enhanced protection when compared to aspirin alone. However, this combination therapy is linked to a higher likelihood of major bleeding, as shown by the ACTIVE W trial, which also indicated that dual antiplatelet therapy provides less protection than warfarin (target international normalized ratio ~2–3) in preventing strokes, systemic embolism, myocardial infarctions, or cardiovascular mortality, while maintaining a similar bleeding risk [31].

In the case of AF patients who undergo percutaneous coronary intervention (PCI) or present with acute coronary syndrome (ACS), there is a recommendation for the use of dual antiplatelet therapy along with OAC. The AUGUSTUS trial demonstrated that adding aspirin to a P2Y12 receptor inhibitor increases the risk of major bleeding (16.1% vs. 9.0%, HR = 1.89, 95% CI: 1.59–2.24, *p* < 0.001) due to the effects of triple therapy. This increased bleeding risk occurred regardless of whether warfarin or apixaban was being utilized, and did not lead to enhancements in death or hospitalization rates (26.2% vs. 24.7%, HR = 1.08, 95% CI: 0.96–1.21, *p* = non-significant) or stroke occurrence (0.9% vs. 0.8%, HR = 1.06, 95% CI: 0.98–1.98) [32]. As a result, it is generally advised that triple therapy be limited to a brief period of less than four weeks for such patients, followed by a regimen that combines a P2Y12 inhibitor with OAC [33].

## 3. Vitamin K Antagonist (VKA)

Until 2010, prevention of AF related stroke had been limited to VKA and antiplatelet agents. Warfarin is a racemic mixture of enantiomers that disrupts the biosynthesis of vitamin K-dependent coagulation factors. Due to the differing half-lives of the various clotting factors, warfarin initially has a pro-thrombotic effect, by blocking proteins C and S before it effectively starts inhibiting activation of coagulation factors II, VII, IX, and X [34]. Due to the initial procoagulant effect, initiation of warfarin often requires administration of a rapid-acting parental anticoagulation agent for the first couple days.

The optimal therapeutic dose of warfarin exhibits considerable variability among patients due to genetic polymorphisms in its receptor, metabolic processes via the cytochrome P450 (CYP) enzyme system, and significant interactions with concomitant medications and dietary factors. Hence, there are significant drawbacks associated with warfarin use, including consistent monitoring to maintain a narrow therapeutic index, measured as prothrombin time in the form of international normalized ratio (INR) [35]. Even though, warfarin has shown to have a 64% reduction in risk of stroke and 26% reduction in mortality in AF patients [24], its use has declined since the advent of direct oral anticoagulants (DOACs) [36], due to its significant drawbacks as mentioned above. On the other hand, warfarin remains the sole therapeutic option for patients with AF and mechanical valves or those with moderate-to-severe mitral valve stenosis, commonly referred to as valvular AF [37, 38].

The European Atrial Fibrillation Trial Study Group concluded that the ideal INR goal should be 3, and values below 2 and above 5 should be avoided [39]. Hence, most patients with AF should maintain an INR of 2.0–3.0 [40]. It is important to measure INR and keep it within the therapeutic range to prevent hemorrhage, which is the most significant adverse effect associated with warfarin use. Warfarin can be reversed with vitamin K, fresh frozen plasma, or prothrombin complex concentrate. One meta-analysis aimed to study the effect of time in therapeutic INR range (TTR) and its effect on stroke risk with warfarin use. It showed the TTR ranged between 25–90% among patients with a mean of 64%. Increasing TTR was linked to a decrease in both major bleeding and stroke risk (*p* < 0.01) [41].

## 4. Direct Acting Oral Anticoagulants

Factor Xa, along with factor Va, facilitates the conversion of prothrombin to thrombin. Thrombin plays a crucial role in the final phase of the coagulation process by transforming fibrinogen into fibrin, thereby forming the thrombus. Dabigatran directly inhibits thrombin, while rivaroxaban, apixaban, and edoxaban serve as inhibitors of factor Xa. The use of DOACs has risen significantly in recent years due to the advantages over warfarin, such as the elimination of INR monitoring and reduced interactions with drugs and food.

Dabigatran was the first DOAC approved by the FDA for AF. The RE-LY trial, a noninferiority study, assessed two doses of dabigatran (110 mg and 150 mg twice daily) against warfarin in AF patients with a CHADS_2_ score over 1. The primary efficacy outcome measured was the incidence of embolic stroke or systemic embolism. Results indicated comparable rates of stroke or embolism for those on the 110 mg dose (relative risk, RR = 0.91; 95% CI: 0.74–1.11; *p* < 0.001 for noninferiority) compared to warfarin, whereas patients on the 150 mg dosage experienced significantly lower rates (RR = 0.66; 95% CI: 0.53–0.82; *p* < 0.001 for superiority). The primary safety outcome, major hemorrhage, was lowest in the 110 mg group (2.71% per year, *p* = 0.003) and similar for both warfarin (3.36% per year) and the 150 mg dabigatran group (3.11% per year, *p* = 0.31). Notably, the annual rate of hemorrhagic stroke was significantly reduced in both dabigatran groups compared to warfarin [42].

The ARISTOTLE trial examined apixaban (5 mg twice daily, or 2.5 mg twice daily for patients meeting at least two of the following criteria: age ≥80 years, weight ≤60 kg, or serum creatinine ≥1.5 mg/dL) in comparison to warfarin (target INR 2.0–3.0). The apixaban regimen showed lower rates of stroke and systemic embolism compared to warfarin (1.27% vs. 1.60% per year; HR = 0.79; 95% CI: 0.66–0.95; *p* < 0.001 for noninferiority; *p* = 0.01 for superiority). Furthermore, apixaban group had significantly lower rates of major bleeding than warfarin (2.13% vs. 3.09% per year; HR = 0.69; 95% CI: 0.60–0.80) [43]. The AUGUSTUS trial also showed that apixaban was linked to a significantly lower risk of major bleeding compared to warfarin, while the stroke rate was also reduced in the apixaban group. The mortality rate, however, was comparable between the two (3.3% vs 3.2%, HR = 1.03, 95% CI: 0.75–1.42) [32].

Rivaroxaban, the first factor Xa inhibitor approved for AF, was studied in the ROCKET AF trial, which randomized 14,264 patients to either rivaroxaban (20 mg/day or 15 mg/day for those with reduced kidney function) or dose-adjusted warfarin. Results indicated that rivaroxaban was non-inferior to warfarin in preventing stroke and systemic embolism (1.7% vs. 2.2%; HR = 0.79, 95% CI: 0.66–0.96). The primary safety endpoint (comprising both major and non-major clinically relevant bleeding) was similar between the two groups, with rivaroxaban showing significantly lower rates of intracranial hemorrhage (0.5% vs. 0.7%; *p* = 0.02) and fatal bleeding (0.2% vs. 0.5%; *p* = 0.003) [44].

The ENGAGE-TIMI 48 trial was a three-arm, randomized controlled study that compared high-dose (60 mg daily) and low-dose (30 mg daily) edoxaban with warfarin in 21,105 AF patients with a CHADS_2_ score greater than 2. Both the edoxaban arms demonstrated noninferiority to warfarin for stroke and systemic thromboembolism prevention. Additionally, both dosages of edoxaban achieved significantly lower rates of major bleeding compared to warfarin [45]. The ELDERCARE-AF study examined the use of very-low-dose edoxaban (15 mg once daily) in 984 Japanese patients aged 80 years and older who had nonvalvular AF and were deemed unsuitable for standard-dose anticoagulation due to a high risk of bleeding or frailty. Edoxaban significantly reduced the risk of stroke or systemic embolism compared to placebo, with an annual event rate of 2.3% for edoxaban versus 6.7% for placebo (HR = 0.34, 95% CI: 0.19–0.61) with no statistically significant difference in major bleeding. Overall, the study suggested that a 15 mg dose of edoxaban provides a favorable balance of efficacy and safety, making it a potential treatment option for frail, elderly patients who are not suitable for standard anticoagulation therapy [46].

A summary of the landmark trials comparing DOAC and Warfarin in AF is provided in Table 3 (Ref. [42, 43, 44, 45]).

**Table 3.** **Summary of landmark trials comparing DOAC and warfarin in atrial fibrillation**.

Stroke/Systemic Embolism						
Trial	DOAC dose studied	N	DOAC (%/y)	Warfarin (%/y)	RR/HR with 95% CI	*p*
RE-LY [42]	Dabigatran 110 mg bd	18,113	1.53	1.69	RR 0.91 (0.74–1.11)	0.34
	Dabigatran 150 mg bd		1.11	1.69	RR 0.66 (0.53–0.82)	<0.001
ROCKET-AF [44]	Rivaroxaban 15–20 mg od	14,264	2.10	2.40	HR 0.88 (0.75–1.03)	0.12
ARISTOTLE [43]	Apixaban 2.5–5.0 mg bd	18,201	1.27	1.60	HR 0.79 (0.66–0.95)	0.01
ENGAGE-AF-TIMI 48 [45]	Edoxaban 60 mg od	21,105	1.57	1.80	HR 0.87 (0.73–1.04)	0.08
	Edoxaban 30 mg od		2.04	1.80	HR 1.13 (0.96–1.34)	0.10
Intracranial Haemorrhage						
Trial	DOAC dose studied	N	DOAC (%/y)	Warfarin (%/y)	RR/HR with 95% CI	*p*
RE-LY [42]	Dabigatran 110 mg bd	18,113	0.12	0.38	RR 0.31 (0.17–0.56)	<0.001
	Dabigatran 150 mg bd		0.10	0.38	RR 0.26 (0.14–0.49)	<0.001
ROCKET-AF [44]	Rivaroxaban 15–20 mg od	14,264	0.50	0.70	HR 0.59 (0.37–0.93)	0.02
ARISTOTLE [43]	Apixaban 2.5–5.0 mg bd	18,201	0.24	0.47	HR 0.51 (0.35–0.75)	<0.001
ENGAGE-AF-TIMI 48 [45]	Edoxaban 60 mg od	21,105	0.26	0.47	HR 0.54 (0.38–0.77)	<0.001
	Edoxaban 30 mg od		0.16	0.47	HR 0.33 (0.22–0.50)	<0.001
Major Bleeding						
Trial	DOAC dose studied	N	DOAC (%/y)	Warfarin (%/y)	RR/HR with 95% CI	*p*
RE-LY [42]	Dabigatran 110 mg bd	18,113	2.71	3.36	RR 0.80 (0.69–0.93)	0.003
	Dabigatran 150 mg bd		3.11	3.36	RR 0.93 (0.81–1.07)	0.31
ROCKET-AF [44]	Rivaroxaban 20 mg od	14,264	3.60	3.40	HR 1.04 (0.9–1.2)	0.58
ARISTOTLE [43]	Apixaban 2.5–5.0 mg bd	18,201	2.13	3.09	HR 0.69 (0.6–0.8)	<0.001
ENGAGE-AF-TIMI 48 [45]	Edoxaban 60 mg od	21,105	2.75	3.43	HR 0.80 (0.71–0.91)	<0.001
	Edoxaban 30 mg od		1.61	3.43	HR 0.47 (0.41–0.55)	<0.001
Total Mortality						
Trial	DOAC dose studied	N	DOAC (%/y)	Warfarin (%/y)	RR/HR with 95% CI	*p*
RE-LY [42]	Dabigatran 110 mg bd	18,113	3.75	4.13	RR 0.91 (0.8–1.03)	0.13
	Dabigatran 150 mg bd		3.64	4.13	RR 0.88 (0.77–1.00)	0.051
ROCKET-AF [44]	Rivaroxaban 20 mg od	14,264	4.50	4.90	HR 0.92 (0.82–1.03)	0.15
ARISTOTLE [43]	Apixaban 2.5–5.0 mg bd	18,201	3.52	3.94	HR 0.89 (0.80–0.998)	0.047
ENGAGE-AF-TIMI 48 [45]	Edoxaban 60 mg od	21,105	3.99	4.35	HR 0.87 (0.79–0.96)	0.08
	Edoxaban 30 mg od		3.80	4.35	RR 0.90 (0.85–0.95)	0.006

bd, twice daily; DOAC, direct oral anticoagulant; HR, hazard ratio; ICH, intracranial hemorrhage; INR, international normalized ratio; od, once daily; RR, relative risk.

Meta-analyses comparing different DOACs with warfarin demonstrated that administration of DOACs was associated with a significant reduction in the risk of stroke/embolism (HR = 0.81), intracranial hemorrhage (HR = 0.48), and all-cause mortality (HR = 0.90), with no significant difference in other bleeding events (HR = 0.86) [47]. In patients with non-valvular AF, the use of DOACs is associated with a 50% lower risk of intracranial hemorrhage and hemorrhagic stroke compared to VKAs [48]. A systematic review of 6 randomized control trials (RCTs) again demonstrated that DOACs were associated with lower all-cause mortality (RR = 0.88, 95% CI: 0.82–0.96) and fatal bleeding rates (RR = 0.60, 95% CI: 0.46–0.77) compared to warfarin, however, DOACs were associated with an increased discontinuation rate due to adverse events (RR = 1.23, 95% CI: 1.05–1.44) [49]. Several other meta-analyses and systematic reviews also revealed more favorable clinical outcomes with DOACs over VKA in patients with non-valvular AF [50, 51]. A meta-analysis of three underpowered trials in patients undergoing electrical cardioversion demonstrated a significantly lower composite incidence of stroke, systemic embolism, myocardial infarction (MI), and cardiovascular death in the DOAC group (0.42%) compared to the warfarin group (0.98%) (RR = 0.42; 95% CI: 0.21–0.86; *p* = 0.017), with no significant difference in major bleeding between the groups [52]. DOACs are contraindicated in certain patient populations; including individuals with mechanical valve replacements or moderate-to-severe mitral stenosis. An increased incidence of both thromboembolic events and major bleeding was observed in patients with mechanical heart valves receiving dabigatran compared to warfarin, resulting in the premature termination of the RCT [38]. Similarly, a trial comparing apixaban to warfarin in patients with mechanical aortic valves was also halted prematurely due to an elevated rate of thromboembolism in the apixaban arm [53]. However, DOACs are not contraindicated in individuals with bioprosthetic heart valves (including mitral valves) or those who have undergone transcatheter aortic valve implantation, where DOAC use has been deemed non-inferior to VKA [54, 55].

In a study of AF patients with rheumatic heart disease, where most had mitral stenosis with a mitral valve area ≤2 cm^2^, warfarin demonstrated a lower risk of cardiovascular events and death compared to rivaroxaban, without a higher risk of bleeding. This finding supports the use of warfarin over DOACs in patients with moderate and severe mitral stenosis [37].

In clinical practice, inappropriate dose reductions of DOACs are often encountered; however, these adjustments should be avoided, as they elevate the stroke risk without significantly mitigating the bleeding risk [56, 57]. Therefore, DOACs should be prescribed at the standard full doses studied in the trials, unless patient meet certain criteria for dose reductions as listed in Table 4.

**Table 4.** **DOAC dose and criteria for dose reductions for AF**.

Drug	Standard Dosing for AF	Dose reduction for AF
Apixaban	5 mg twice daily	∙ Reduce to 2.5 mg twice daily for any 2 of the following
		∙ age ≥80, weight ≤60 kg, or SCr ≥1.5 mg/dL
		∙ or when co administered with combined P-gp and CYP3A4 inhibitors
Rivaroxaban	20 mg once daily with food	∙ Reduce to 15 mg daily for CrCl ≤50 mL/min
Edoxaban	60 mg once daily	∙ Reduce to 30 mg once daily for for CrCl 15–50 mL/min
		∙ Contraindicated if CrCl >95 mL/min due to increased ischemic stroke risk compared to warfarin
		∙ Contraindicated if CrCl <15 mL/min or on dialysis
Dabigatran	150 mg twice daily	∙ Reduce to 75 mg twice daily for CrCl 15–30 mL/min or for CrCl 30–50 mL/min with concomitant dronedarone or ketoconazole use
		∙ Contraindicated if CrCl <15 mL/min or on dialysis

SCr, serum creatinine; CrCL, creatinine clearance; CYP3A4, cytochrome P450 3A4 enzyme.

In AF patients with CKD, warfarin use was linked to an increased risk of hemorrhagic stroke [56]. DOACs remain more efficacious and safe when compared to VKA in mild to moderate CKD (creatinine clearance >30 mL/min) [57]. Dose-adjusted apixaban has shown to reduce the risks of bleeding, embolism, and death compared to warfarin in CKD patients [58, 59]. In cancer patients, the traditional CHA₂DS₂-VASc score is not deemed useful as cancer is a hypercoagulable state and patients often have altered hemostasis. Recently DOACs have become a preferable choice because of data supporting their efficacy in cancer patients [58]. In patients with impaired liver function, DOACs show promise because they depend less on liver metabolism compared to warfarin, which could potentially lead to greater safety. Observational studies indicate that DOACs may reduce the risks of major bleeding and mortality in this population while still effectively preventing thromboembolism [60]. However, it is crucial to carefully consider advanced hepatic fibrosis/liver cirrhosis when stratifying risk for anticoagulation management. Recent evidence supports the use of DOACs in patients with chronic liver disease (CLD) who do not have cirrhosis and those classified as Child-Pugh A, but DOACs are not recommended for those classified as Child-Pugh B or C.

The lack of specific reversal agents for DOACs was previously regarded as a significant disadvantage for DOACs in comparison to warfarin. However, the approval of idarucizumab, a monoclonal antibody, by the FDA for the reversal of dabigatran addressed this concern [59]. Subsequently, in 2018, the FDA approved andexanet alfa, a recombinant modified Factor Xa protein, for the reversal of rivaroxaban and apixaban in cases of life-threatening bleeding [61].

Notably, to date, no randomized controlled trials have directly compared different DOACs. However, a systematic review found that dabigatran had a lower risk of stroke or systemic embolism compared to rivaroxaban and edoxaban, with outcomes similar to apixaban. Major bleeding rates were comparable between apixaban and edoxaban, and lower than those seen with dabigatran and rivaroxaban [62].

## 5. Left Atrial Appendage Occlusion (LAAO)

Left atrial appendage occlusion has recently emerged as a method for preventing stroke in patients who cannot tolerate oral anticoagulation. The PROTECT AF and PREVAIL trials evaluated the efficacy and safety of the LAAO closure device Vs warfarin [63, 64]. These two trials found that LAAO was non inferior for the primary end point (stroke, systemic embolism, cardiovascular/unexplained death) compared to warfarin. The PRAGUE-17 trial, demonstrated the non-inferiority of LAOO (HR: 0.84; 95% CI: 0.53–1.31; *p* = 0.44) when compared to DOACs for its primary end points (stroke, TIA, CV death, major or nonmajor clinically relevant bleeding, or procedure-/device-related complications) [65]. Data from these trials led to the FDA approval of LAAO devices in 2015. However, the class of recommendation is weak because of low level of evidence at this time [66], but the evidence is rapidly evolving.

We have summarized the current ESC [67] and ACC/AHA [68] guidelines pertaining to thromboembolic risk assessment and OAC for AF in Tables 5,6.

**Table 5.** **ESC guidelines for thromboembolic risk assessment and oral anticoagulation**.

Recommendations for assessment of stroke risk in AF		
Class	Level	Recommendation
I	A	Oral anticoagulants are advised in patients with elevated thromboembolic risk
I	C	CHA_2_DS_2_-VA score ≥2 denotes an elevated thromboembolic risk for OAC initiation
I	B	All patients with hypertrophic cardiomyopathy irrespective of CHA_2_DS_2_-VA score should receive OAC
I	B	Periodic reassessment of thromboembolic risk for the appropriateness of OAC
IIa	C	Class IIa recommendation to initiate OAC for CHA_2_DS_2_-VA score of 1
IIb	B	OAC could be considered for those with asymptomatic AF at an elevated thromboembolic risk
III	A	Antiplatelet is not an appropriate substitute for anticoagulation.
Recommendations for oral anticoagulant use in AF		
I	A	DOACs are preferred over VKAs (not in mechanical heart and moderate or severe mitral stenosis).
I	B	On VKA, maintain a goal INR of 2–3
I	B	Transitioning to a DOAC is recommended for those with inadequate time in therapeutic range while on warfarin therapy (TTR <70%).
IIa	A	Maintaining a TTR >70% for VKA users is advisable.
IIb	B	For patients aged 75 or older on a stable VKA regimen, continuing VKA may be preferred over substituting VKA with DOAC, due to bleeding risks.
III	B	DOAC dose should not be reduced, unless patients meet specific criteria for dose reduction

OAC, oral anticoagulation; TTR, time in therapeutic range; VKA, vitamin K antagonist.

**Table 6.** **ACC/AHA guidelines for thromboembolic risk assessment and oral anticoagulation**.

Recommendations for Stroke risk		
Class	Level	Recommendations
1	B-R	In patients with AF having a ≥2% annual risk of stroke, OAC is recommended.
1	B-NR	Periodic reevaluation of the need for and choice of anticoagulation therapy is recommended
Recommendations for Antithrombotic Therapy		
1	A	AF and an annual thromboembolic risk of ≥2% (i.e., CHA_2_DS_2_-VASc score ≥2 for men, ≥3 for women) should receive OAC to prevent stroke.
1	A	In those with no rheumatic mitral stenosis or mechanical heart valves, use DOACs over warfarin.
2a	A	For those with AF with a thromboembolic risk of ≥1% but <2% (CHA_2_DS_2_-VASc score 1 for men, 2 for women), OAC is reasonable.
3: Harm	B-R	AF patients eligible for anticoagulation should not use aspirin alone or with clopidogrel as an alternative to anticoagulation for stroke risk reduction.
3: No Benefit	B-NR	Aspirin monotherapy in AF patients without stroke risk factors provides no benefit.
Recommendations for Managing Anticoagulants		
1	C-LD	For AF patients on DOACs, manage drug interactions carefully, especially with CYP3A4 and/or P-glycoprotein modifiers.
1	B-R	For AF patients on warfarin, maintaining a target INR of 2–3 is recommended with routine INR checks.
3: Harm	B-NR	Nonevidence-based doses or reduced doses of DOACs should be avoided.

B-R, Moderate-quality evidence from randomized trials; B-NR, moderate-quality evidence from nonrandomized studies; C-LD, limited data from observational or registry studies.

## 6. Conclusion 

Atrial fibrillation management necessitates a comprehensive approach to stroke prevention, primarily through anticoagulation therapy. Given the increasing prevalence of AF and its associated morbidity and mortality, effective strategies are critical in mitigating the risk of thromboembolic events. This review outlines the current recommendations and provides an overview of the literature on stroke prevention in atrial fibrillation. DOACs have emerged as safer and more effective alternatives to traditional therapies like warfarin, due to improved ease of use, predictable pharmacokinetics, and reduced need for extensive monitoring. The shift toward DOACs has drastically transformed clinical practice, especially for patients at an increased risk of bleeding. Non-pharmacological strategies, such as left atrial appendage closure devices, provide promising options for patients at high risk of stroke who may not tolerate long-term anticoagulation.
